# Non‐Metal Ion Storage in Zinc‐Organic Batteries

**DOI:** 10.1002/advs.202310319

**Published:** 2024-03-13

**Authors:** Ziyang Song, Ling Miao, Yaokang Lv, Lihua Gan, Mingxian Liu

**Affiliations:** ^1^ Shanghai Key Lab of Chemical Assessment and Sustainability School of Chemical Science and Engineering Tongji University Shanghai 200092 P. R. China; ^2^ College of Chemical Engineering Zhejiang University of Technology Hangzhou 310014 P. R. China

**Keywords:** charge storage mechanisms, energy storage, non‐metal ions, organic electrodes, zinc‐organic batteries

## Abstract

Zinc‐organic batteries (ZOBs) are receiving widespread attention as up‐and‐coming energy‐storage systems due to their sustainability, operational safety and low cost. Charge carrier is one of the critical factors affecting the redox kinetics and electrochemical performances of ZOBs. Compared with conventional large‐sized and sluggish Zn^2+^ storage, non‐metallic charge carriers with small hydrated size and light weight show accelerated interfacial dehydration and fast reaction kinetics, enabling superior electrochemical metrics for ZOBs. Thus, it is valuable and ongoing works to build better ZOBs with non‐metallic ion storage. In this review, versatile non‐metallic cationic (H^+^, NH_4_^+^) and anionic (Cl^−^, OH^−^, CF_3_SO_3_^−^, SO_4_^2−^) charge carriers of ZOBs are first categorized with a brief comparison of their respective physicochemical properties and chemical interactions with redox‐active organic materials. Furthermore, this work highlights the implementation effectiveness of non‐metallic ions in ZOBs, giving insights into the impact of ion types on the metrics (capacity, rate capability, operation voltage, and cycle life) of organic cathodes. Finally, the challenges and perspectives of non‐metal‐ion‐based ZOBs are outlined to guild the future development of next‐generation energy communities.

## Graphical abstract

An overview of versatile non‐metallic cationic and anionic charge carriers in zinc‐organic batteries is demonstrated, with a comparison of their respective properties and chemical interactions with redox‐active organic materials, giving insights into the impact of ion types on electrochemical performances. Current challenges and outlooks of non‐metal‐ion‐based zinc batteries are outlined to guide future development of next‐generation energy realms.

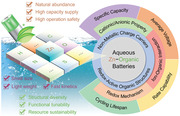

## 1 Introduction

With the increasing energy crisis and environmental pollution issues, there is an urgent need to exploit efficient and sustainable energy storage systems to build a greener world.^[^ ^1^ ^]^ Lithium‐ion batteries as a typic power source have dominated the energy industry with great success in various uses of portable electronics and new energy vehicles.^[^ ^2^ ^]^ However, potential safety issues, scarce lithium resources and rising costs limit their further development.^[^ ^3^ ^]^ Therefore, developing advanced battery systems beyond lithium‐ion storage is of great significance for propelling energy storage. Aqueous zinc‐organic batteries (ZOBs) have recently inspired numerous interests in energy realms due to their natural sustainability, affordability, and avoidance of explosion and fire risks.^[^ ^4^ ^]^ ZOBs involve Zn metal anodes with high capacity and low Zn/Zn^2+^ redox potential, redox‐active organic cathodes with structural and functional designability, and safe aqueous electrolytes, which are reactors for chemical redox reactions between charge carriers and hosting electrodes in water media.^[^ ^5^ ^]^ In particular, Zn^2+^ plating/stripping reaction allows the high compatibility of Zn anodes with organic cathodes in aqueous electrolytes,^[^ ^6^ ^]^ making ZOBs an attractive candidate for advanced grid‐scale energy storage.

The redox chemistries and performance metrics of ZOBs are largely determined by ion migration inside the electrodes, which in turn depend on the selection of ionic charge carriers.^[^ ^7^ ^]^ So far, most attention has been focused on the operation of metal‐ion batteries,^[^ ^8^ ^]^ but relatively little consideration has been given to searching for non‐metallic ionic carriers. Conventionally, metallic Zn^2+^ cations are demonstrated as charge carriers in ZOBs, but their large hydrated configuration and high diffusion barrier often induce sluggish interfacial charge conveying kinetics.^[^ ^9^ ^]^ Besides, the repeated insertion/desertion of Zn^2+^ ions easily cause damage to the electrode structure, especially at high current rates. Non‐metallic charge carriers such as H^+^ and NH_4_^+^ provide significant advantages beyond conventional Zn^2+^ reaction, including resource abundance, high migration rate and low reaction energy roadblock.^[^ ^10^ ^]^ If the uptake/removal of non‐metallic ions can be achieved in organic cathodes, the energy metrics of ZOBs will be substantially reformed, and the horizons of battery chemistry can be also broadened. Up to now, various non‐metallic charge carriers have been fulfilled to couple with organic materials, which can be categorized into cationic (e.g., H^+^, NH_4_^+^) and anionic (e.g., Cl^−^, OH^−^) species based on their charges.^[^ ^11^ ^]^ Owing to the distinct redox reactions between ionic carriers and organic materials, non‐metal‐ion ZOBs delivers different electrochemical properties and charge storage mechanisms compared to Zn^2+^‐based ones, which is a crucial consideration for the construction of high‐performance ZOBs. However, a comprehensive understanding of the relationship between non‐metal charge carriers and organic cathode materials in ZOBs is still limited.

**Figure 1:**
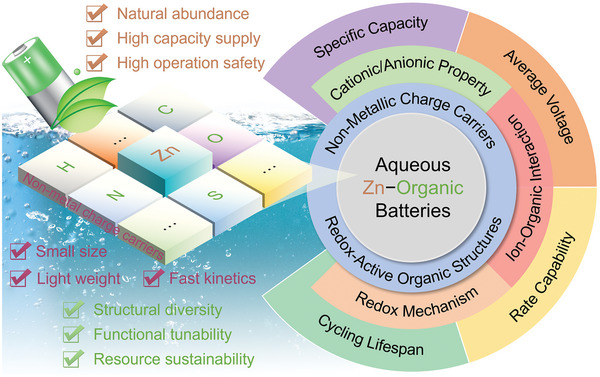
Non‐metallic charge carrier storage in aqueous Zn‐organic batteries.

In this review, we strive to give an overview of non‐metallic charge carrier storage in ZOBs (**Figure** **1**). The non‐metallic ions are first summarized and categorized with a schematic comparison of their respective features and redox interactions with organic materials. Then, recent advances in emerging organic cathodes are exhibited with an emphasis on the (de)coordination reactions between non‐metallic cationic/anionic charge carriers and active functionalities. We highlight the effects of non‐metallic ionic species on the electrochemical metrics (capacity, rate capability, redox voltage and cyclic stability) of organic cathode materials, giving insights into the structure–property relationship for building better ZOBs. Finally, the challenges and outlooks of ZOBs in terms of design principles and applications are outlined to guide the future development of next‐generation energy‐related communities, achieving a greener rechargeable world.

## 2 Overview of Non‐Metallic Charge Carriers

### 2.1 Properties of Ionic Carriers

**Figure 2:**
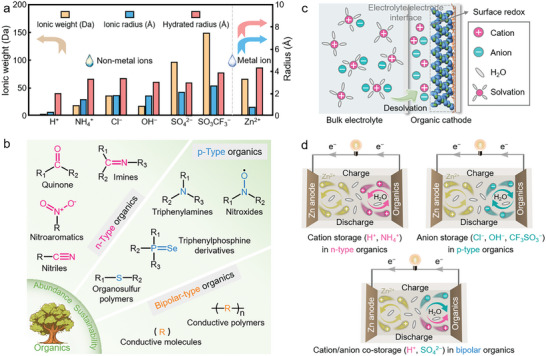
a) Comparison of physicochemical properties of non‐metallic charge carriers. b) Structural design of organic cathode materials. c) Schematic desolvation of non‐metallic ions at the electrolyte/electrode interface. d) Redox charge storage mechanisms of ZOBs.

The selection of charge carriers is one of the key factors determining electrochemical metrics. As a typical ion‐storage mechanism, Zn^2+^ uptake/removal often occurs in organic cathodes, accompanied by the co‐storage of non‐metal ions (e.g, H^+^, CF_3_SO_3_^−^).^[^ ^3^ ^]^ Of note, Zn^2+^‐storage electrochemistry is characterized by slow reaction kinetics, mainly due to the high desolvation and coordination energies demanded for the uptake of hydrated large‐sized Zn^2+^ ions into organic hosts.^[^ ^11^ ^]^ In contrast, the small and light non‐metallic charge carriers deliver high migration rate and low reaction energy barriers,^[^ ^11^ ^]^ which are prior to Zn^2+^ ions into redox‐sites and dominate the energy storage for fast and stable ZOBs. To date, abundant studies have been carried out on the creation of ZOBs involving a variety of non‐metallic ionic charge carriers, which can be categorized into cations (H^+^, NH_4_^+^) and anions (Cl^−^, OH^−^, CF_3_SO_3_^−^, SO_4_^2−^) based on their charges (**Figure** **2**a).

Non‐metal‐ion charge carriers are composed of Earth‐rich elements and have less corrosiveness, making them cost‐effective and safe, especially in large amounts. Among these ions, protons (H^+^) have received much attention due to the advantages:^[^ ^12^ ^]^ i) H element is richly available, affordable and sustainable; ii) H^+^ shows the lowest atomic weight (1 g mol^−1^) and the smallest ionic radius (2.28 Å), which can achieve ultrafast electrochemical reaction kinetics; iii) H^+^ can also reduce the whole weight of batteries to meet the high‐energy‐power requirements for large‐scale energy storage. However, most studies about ZOBs focus on Zn^2+^ storage, accompanied by the co‐insertion of H^+^ ions due to the weak acidity of electrolytes. Thus, the improvement of H^+^ storage is in highly demand to substantially reform the performances of ZOBs, but remains in its infancy.

Although resourceful H^+^ reaction is applied for ZOBs, strong acidic electrolytes would result in corrosion to the electrodes and current collectors, leading to inferior electrochemical stability.^[^ ^13^ ^]^ Furthermore, the potential leakage of acidic solutions would cause environmental pollution. In contrast, nontoxic, mild and safe NH_4_^+^ electrolytes for the operation of ZOBs offer several great advantages:^[^ ^14^ ^]^ i) the ultralow molar mass (18 g mol^−1^) and small hydrated ionic radius (3.31 Å) of NH_4_^+^ charge carrier does a favor to fast diffusion kinetics and accelerated dehydration; ii) the hydrolysis of ammonium salts helps to establish a neutral or weakly acidic electrolyte environment, which is much less corrosive and thus suppresses side reactions; iii) NH_4_^+^ shows tetrahedral shape and powerful preferential orientation, and their non‐metallic H‐bonding interaction with electrodes is quite flexible compared with rigid Zn^2+^ metallic bonds, triggering a strong vitality to overcome the kinetics and stability obstacles. All these superiorities make NH_4_^+^ ions promising alternatives for next‐generation ZOBs.

Compared with non‐metallic cations, non‐metallic anions generally possess higher molar masses and ionic sizes, which result in different battery chemistries and performances.^[^ ^11^, ^15^ ^]^ Anion storage in ZOBs is generally characterized by diffusion‐controlled slow kinetics, and structural variations caused by formation/fracture cycles of covalent ionic bonds result in pulverization effect in electrodes. Reports on ZOBs with Cl^−^, OH^−^, CF_3_SO_3_^−^, and SO_4_^2−^ storage have confirmed the key impact of anions on electrochemical performances,^[^ ^7a^ ^]^ imposing stricter requirements for rational design of electrode materials. In fact, various anionic and cationic species in electrolyte all contribute to electrochemical energy storage behaviors, as illustrated in dual‐ion batteries. Paying more attention to the anion‐electrode compatibility during the electrochemical process, which is expected to effectively propel the development of ZOBs.

Except for charge carriers, electrode materials are fundamental components of batteries. Due to natural sustainability, structural diversity and functional tunability, π‐conjugated aromatic organic materials are exhibited to be promising redox‐active cathode candidates for rechargeable ZOBs.^[^ ^16^ ^]^ Generally, the energy storage behaviors in ZOBs depend on the surface coordination reactions between ionic charge carriers of electrolytes and redox‐active motifs of organic cathodes.^[^ ^17^ ^]^ The surface chemistry, although crucial, determines the charge migration/storage kinetics of organic cathodes in electrochemistry‐sensitive processes only if that chemistry is accessible for electrolyte ions.^[^ ^18^ ^]^ The structural design of organic cathode materials is thus crucial for ZOBs. Up to now, there have been some preliminary investigations on non‐metallic ion storage in organic materials.^[^ ^19^ ^]^ Based on the types of redox‐active groups and their charge‐state alteration during the electrochemical process (Figure 2b), the reported organic cathode materials in ZOBs can be divided into three types: n‐type (storing cations), p‐type (storing anions), and bipolar‐type (co‐storing cations/anions). The n‐type organic materials often show high‐density cation‐coordination active sites to provide high capacities, including quinones, imines, nitroaromatics and nitrile compounds.^[^ ^20^ ^]^ Different from n‐type counterparts, p‐type organic materials typically possess high voltage output, entailing triphenylamine derivatives, nitroxide compounds, triphenylphosphine derivatives and organosulfur compounds.^[^ ^21^ ^]^ Bipolar‐type organic materials combine the respective favorable features of n‐type and p‐type cases, including conductive molecules and polymers such as polyaniline, polypyrrole and polythiophene skeleton.^[^ ^22^ ^]^ Profiting from the multiplicity of molecular structures, there is a broad development space to design redox‐active organic materials to fulfill the requirements of ZOBs with high performances in the future.

### 2.2 Ion‐Organic Interactions

Non‐metallic‐ion ZOBs with different redox behaviors have exhibited their competitiveness and prospect to replace metal‐ion systems. The importance of electrode‐electrolyte interaction in ZOBs is receiving attention, as it strongly affects the stability and durability of surface redox reactions. Non‐metallic charge carriers interact with organic cathode materials in their (de)solvated states (Figure 2c). The solvation behaviors of electrolyte ions generally have two significant impacts:^[^ ^11c^ ^]^ i) ion‐migration kinetics in bulk electrolytes and ii) thermodynamic desolvation process at the electrolyte/electrode interface. Compared with metallic Zn^2+^ ions, the low‐solvation architecture of non‐metallic ions is advantageous for rapid reaction kinetics and structural stability. Desolvation is a nonspontaneous and energy‐consuming procedure, which demands extra desolvation energy to strip ionic carriers from H_2_O solvation sheath.^[^ ^11a^ ^]^ Ionic carriers are often de‐solvated at the electrolyte/electrode interface to provide hydrated or net ions for redox reactions with organics. Therefore, the interfacial reactions determine the (dis)charge rate capability and can be the rate‐limiting step during battery operation.

For example, H_3_O^+^ can be inserted into organic electrode hosts instead of H^+^, as the large dehydration energy barrier (11.66 eV) of H_3_O^+^ causes it to remain solvated state in aqueous solution.^[^ ^23^ ^]^ Recent studies have discovered that hydrated NH_4_^+^(H_2_O)_6_ ions with loose solvation frameworks deliver easier desolvation process to afford a net state for accelerating interfacial diffusion/reaction kinetics due to the synergy of flexible H‐bonds and low desolvation energy barriers.^[^ ^11^, ^24^ ^]^ Of note, there have been no reports of other non‐metallic charge carriers completing the insertion process in their hydrated form. The magnitude of the desolvation energy is governed by the Stokes radius, where the high charge density of net ions results in a high energy barrier for desolvation process.^[^ ^11^, ^25^ ^]^ The low desolvation energy barrier is associated with the low energy penalty for high discharge potentials, allowing easy access to net ions for fast and stable electrochemical response. Charge‐neutral H_2_O molecules can spontaneously diffuse and achieve dynamic equilibrium between bulk electrolytes and electrodes, thereby determining the migration direction of solvated ions and cyclic stability through H_2_O activity.^[^ ^11^, ^26^ ^]^ Therefore, rational solvation design of aqueous charge carriers is crucial for modulating the electrode‐electrolyte interactions to afford efficient ZOBs.

### 2.3 Redox Charge Storage Mechanisms

The electrochemical performances of ZOBs depend on their energy storage mechanisms involving different chemical interactions between non‐metal charge carriers and organic cathodes. During battery operation, the Zn anode experiences the reversible Zn^2+^ plating/stripping reaction, which makes it highly compatible with organic cathodes. Generally, the charge storage behaviors of ionic carriers in organic cathodes can be categorized into three types (Figure 2d and **Table** **1**):^[^ ^27^ ^]^ cation storage in n‐type organic cathodes, anion storage in p‐type organic cathodes, cation/anion co‐storage in bipolar‐type organic cathodes. During the electrochemical reaction process, n‐type organics first undergo a reduction reaction to generate a negatively charged state by accepting electrons.^[^ ^22d^ ^]^ Subsequently, these anionic sites combine with cationic carriers to maintain the charge neutral. In such a procedure, the use of non‐metallic cations relies on the molecular structures of n‐type organics. Although most n‐type organic cathodes that store cations (e.g., H^+^, NH_4_^+^) show high capacity, their low operating potential (<0.8 V versus Zn/Zn^2+^) is an inherent drawback.^[^ ^28^ ^]^ Conversely, p‐type organics first experience an oxidation process to donate electrons and convert into positively charged cations, which then react with non‐metallic anions (e.g., Cl^−^, OH^−^, CF_3_SO_3_^−^) to balance the charge. Despite the high redox potential and rapid kinetics, p‐type organic cathodes generally suffer from low capacity (<200 mAh g^−1^) due to the majority of redox‐inactive structures.

**Table 1:** A comparison of various organic materials and charge carriers in ZOBs.

Organics	Active groups	Charge carriers	Advantages	Disadvantages
n‐type	C═O, C═N, C≡N, NO_2_	H^+^, NH_4_^+^	High activity; High capacity	Low voltage (<0.8 V)
p‐type	NH, NR, NO•, ─S─, P═Se	Cl^−^, OH^−^, CF_3_SO_3_^−^	High voltage	Low capacity (<150 mAh g^−1^)
Bipolar	C═N/NH; C═O/─S─	H^+^/SO_4_^2−^	Improved voltage; Extended stability	Insufficient capacity (<200 mAh g^−1^)

Bipolar‐type organics can simultaneously act as electron acceptors and donors, which can be either reduced or oxidized to achieve alternative storage of opposite charges (e.g., H^+^, SO_4_^2−^).^[^ ^29^ ^]^ In general, bipolar‐type organics combine high‐capacity n‐type and high‐voltage p‐type redox sites in an extended organic skeleton, which exert the synergetic benefits of n/p‐type mixing reaction mechanism and delivers high structural stability for robust electrochemical reactions. However, bipolar organic materials are still in their early stage, which suffer from insufficient capacity (<200 mAh g^−1^) because of the low‐density redox‐active sites in n/p‐fused structures. Therefore, more efforts should be made to develop multielectron bipolar‐type redox organics for building advanced ZOBs.

## 3 Implementation Effect of Non‐Metallic Charge Carriers

### 3.1 Non‐Metallic Cation Storage

#### 3.1.1 H^+^ Ions as Charge Carriers

Compared with Zn^2+^ reaction, fast‐kinetics H^+^ ions are preferentially considered as charge carriers to propel ZOBs.^[^ ^30^ ^]^ The participation of protons in aqueous electrolytes can endow organic electrodes with a higher capacity than that in proton inertia media (neutral or organic electrolytes).^[^ ^31^ ^]^ Thus, boosting the competitiveness of H^+^ storage against Zn^2+^ is anticipated to reform the performances of ZOBs. In this regard, owing to the inevitable presence of H^+^ in mild aqueous electrolyte such as ZnSO_4_ and Zn(CF_3_SO_3_)_2_ solutions (with a pH value of 4–5), H^+^ can react with redox‐active groups (e.g., imine, carbonyl, azo) of organic compounds before or in parallel with Zn^2+^ storage through the dynamic hydrolysis reaction of Zn salt byproducts. It is thus highly desired to develop advanced and safe ZOBs in mild electrolytes.

**Figure 3:**
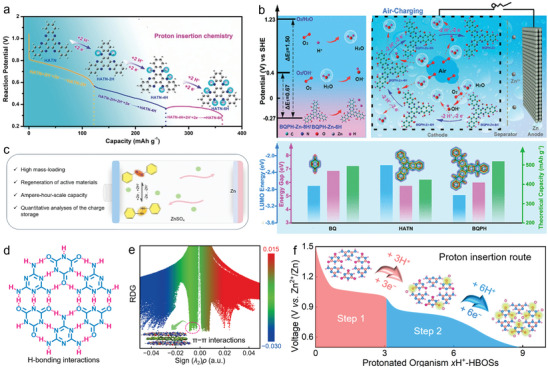
a) Proton‐coupled structural evolution of HATN molecule. Reproduced with permission.^[^ ^17^ ^]^ Copyright 2020, Wiley‐VCH. b) Energy level transition diagram and operation mechanism of BQPH, and parameter comparison of different organic molecules. Reproduced with permission.^[^ ^32^ ^]^ Copyright 2022, American Chemical Society. c) Illustrations of a recyclable and scalable OEM for ZOBs. Reproduced with permission.^[^ ^35^ ^]^ Copyright 2023, Wiley‐VCH. d) Lock‐key H‐bonded 2D supramolecular planar structure. e) Density gradient plots and gradient isosurface. f) Two‐step H^+^ insertion in HBOSs cathode during discharging. Reproduced with permission.^[^ ^39^ ^]^ Copyright 2023, Wiley‐VCH.

Niu group first reported pure H^+^ insertion chemistry of π‐conjugated aromatic diquinoxalino [2,3‐a:2′,3′‐c] phenazine (HATN) cathode in mild ZnSO_4_ electrolyte (**Figure** **3**a).^[^ ^17^ ^]^ Different from previously reported Zn^2+^ storage mechanism in ZOBs, high‐kinetics H^+^ uptake/removal was prior to Zn^2+^ into protophilic pyrazine nitrogen sites via a three‐step six‐electron redox reaction. The reversible precipitation/dissolution of Zn_4_SO_4_(OH)_6_·5H_2_O product on HATN cathode accounted for H^+^ uptake/removal during the electrochemical process. Such a proton insertion electrochemistry endowed HATN cathode with ultrahigh capacities (405 mAh g^−1^ at 0.1 A g^−1^ and 123 mAh g^−1^ at 20 A g^−1^). This work would broaden the electrochemical horizons of ZOBs and give new avenues to design high‐performance organic cathode materials.

With a conjugated molecular structure similar to HATN, benzo[i]benzo[6,7]quinoxalino[2,3‐a]benzo[6,7]‐quinoxalino[2,3‐c]phenazine‐5,8,13,16,21,24‐hexaone (BQPH) was recently reported as the cathode for air‐rechargeable ZOBs with H^+^ coordination chemistry in mild ZnSO_4_ electrolyte (Figure 3b).^[^ ^32^ ^]^ During the operation of Zn||BQPH battery, the primary coordination of a Zn^2+^ ion with adjacent C═O/C═N groups leaded to uneven charge distribution in BQPH skeleton, which triggered subsequent H^+^ insertion on the residual four pairs of C═O/C═N motifs. Intriguingly, the large voltage discrepancy between oxygen and discharged BQPH cathode induced a spontaneous redox reaction between both, of which BQPH was oxidized by oxygen in the air. The potential of BQPH cathode gradually increased along with H^+^ extraction, and the discharged cell was air‐recharged without an external power source. As a result, a five‐step 10e^−^ H^+^‐dominant coordination with BQPH showed a high capacity of 351 mAh g^−1^ at 0.1 A g^−1^ and a repeated self‐recharge ability. This study would give valuable understanding about the H^+^ storage mechanism of ZOBs and broaden the horizons of organic materials.

Currently reported organic materials for ZOBs usually focused on single‐electron‐acceptor functionalities (e.g., C═O, C═N), which showed limited H^+^ transfer reactions.^[^ ^13^, ^23^, ^33^ ^]^ Thus, exploring new functionalities with multi‐electron‐acceptor capability is promising to build high‐capacity ZOBs. Our group recently demonstrated redox‐active dinitrobenzene molecules containing two successive two‐electron processes, as the cathode materials for ZOBs.^[^ ^34^ ^]^ Benefiting from strong redox activity, *para*‐dinitrobenzene hosted in carbon flowers harvested a large capacity of 402 mAh g^−1^ and a superb stability up to 25 000 cycles. This work would open new paths for designing multi‐electron nitroaromatics, but the research of nitro electrochemistry is still in its infancy. Besides, Wang group reported a low‐cost and recyclable azobenzene cathode (OEM) for ZOBs (Figure 3c).^[^ ^35^ ^]^ The azo group of azobenzene could take place 2e^−^ H^+^‐coordination reaction to afford ZOBs with high capacity even under high mass loadings of active materials. The assembled four‐layer laminated pouch cell exhibited Ampere‐hour‐scale capacity (0.5 Ah, 7.8 mAh cm^−2^) and cycling stability (200 cycles). Thanks to the small molecule properties and stable (dis)charged states, azobenzene cathode can be recycled with a high yield (over 90%) at any charging state. This work represents a substantial step in the practical uses of organic materials for recyclable and scalable ZOBs.

In addition to the size advantage of H^+^ ions, the presence of H as a constituent of H_2_O enables rapid proton diffusion kinetics with unparalleled mobility in aqueous environments by a displacive mechanism first postulated by von Grotthuss.^[^ ^36^ ^]^ This motion is similar to Newton's cradle, where the associated local displacement leads to the long‐range proton transfer, which is completely different from the long‐distance conduction of discrete metal ions.^[^ ^37^ ^]^ Previous investigations have confirmed that Grotthuss proton transfer can also occur in H‐bonding solid networks and metallic oxide electrode.^[^ ^38^ ^]^ Therefrom, the originality of Grotthuss proton mechanism inspires us to consider whether Grotthuss topochemistry can be fulfilled in H‐bonded organic electrode materials to unlock the intrinsic limitations of battery chemistries, thereby creating ZOBs with large‐current tolerance and cyclability.

Most recently, our group reported the supramolecular self‐assembly of 1,3,5‐triazine‐2,4,6‐triamine and cyanuric acid into H‐bonded organic superstructures (HBOSs) through H‐bonding in plane and π‐π interaction out‐of‐plane (Figure 3d,e).^[^ ^39^ ^]^ Flower‐shaped and ultrastable superstructures inhibited their dissolution in aqueous electrolytes and provided rich interaction interface for activating redox‐sites. The lock‐and‐key H‐bonding networks of HBOSs allowed the rapid dance of protons along with the concerted formation/cleavage of H‐bonds to deeply access protophilic C═O sites through a two‐step 9e^−^ redox reaction (Figure 3f). This facile proton relay mode stimulated the redox reaction of HBOSs cathode to enable the state‐of‐the‐art ZOBs with high‐rate capability (135 mAh g^−1^ at 150 A g^−1^), high energy supply (267 Wh kg^−1^) and excellent lifespan (50 000 cycles at 10 A g^−1^). Follow these findings, Zhu group further designed a H‐bonded supramolecular organic framework cathode (HOF‐HATN) derived from diaminotriazine‐decorated hexaazatrinnphthalene for rapid Grotthuss H^+^ conduction toward efficient ZOBs.^[^ ^31b^ ^]^ These researches confirm that H‐bonding organic networks with rapid Grotthuss proton conduction is particularly suitable for the construction of ZOBs with superior H^+^ storage, but there are few considerations in this regard.

#### 3.1.2 NH_4_^+^ Ions as Charge Carriers

In NH_4_^+^ ions, the nitrogen atom is situated the center and surrounded by four terminated hydrogen atoms, which serve as binding atoms to the structural oxygen/nitrogen atoms of organic electrode frameworks through H‐bonding interactions (N─H─O and N─H─N).^[^ ^14^, ^40^ ^]^ Similar to H^+^ storage, the reactions between NH_4_^+^ ions and organic electrodes follow the donor–acceptor principle,^[^ ^41^ ^]^ which contributes to more electron‐accommodating sites compared to metal ion storage. Compared with high‐kinetics H^+^ ions, the implementation of polyatomic NH_4_^+^ charge carrier has shown superior electrochemical stability, due to the formation of anti‐dissolvable flexible H‐bonded networks. However, due to the relatively larger radius of NH_4_^+^ ions, their reaction kinetics are slightly lower than that of H^+^ ions, and more structural space is required for NH_4_^+^ accommodation. Besides, NH_4_^+^ electrolytes show mild acidity and less corrosiveness, and do not cause material dissolution.

**Figure 4:**
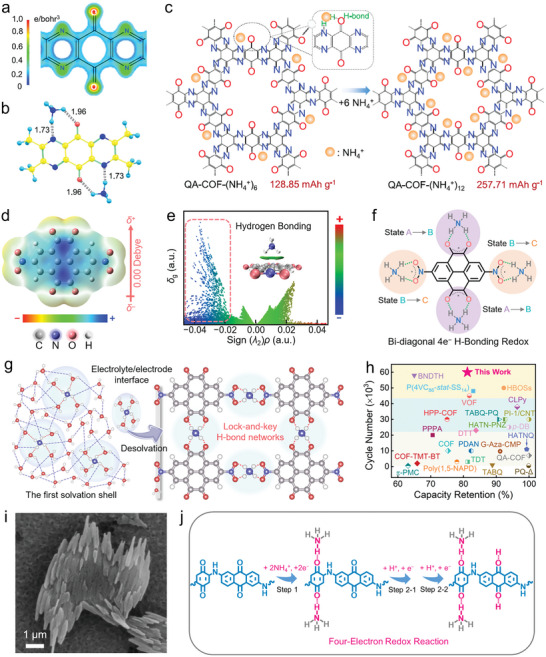
a) Molecular structure and b) bi‐diagonal two‐coordination configuration with two NH_4_^+^ ions. c) The six‐ and twelve‐coordination structures of QA‐COFs. Reproduced with permission.^[^ ^11c^ ^]^ Copyright 2021, American Chemical Society. d) Molecular electrostatic potentials, e) scatter plot and gradient isosurface of the independent gradient model of DNPT. f) H‐bonding redox mechanism of DNPT cathode during discharging. g) Illustration of NH_4_^+^ desolvation at the electrolyte/electrode interface. h) Lifespan of DNPT cathode compared with reported organics. Reproduced with permission.^[^ ^24^ ^]^ Copyright 2023, Wiley‐VCH. i) SEM image of OSs and j) corresponding redox reaction process. Reproduced with permission.^[^ ^44^ ^]^ Copyright 2023, Wiley‐VCH.

There have been some preliminary studies on NH_4_^+^ storage, which give appealing insights into the rational design of NH_4_^+^‐hosting organic materials.^[^ ^42^ ^]^ Alshareef group reported a distinctive H‐bond electrochemistry to utilize a covalent organic framework (QA‐COFs) for NH_4_^+^ storage (**Figure** **4**a,b).^[^ ^11c^ ^]^ The unique coordination behaviors of non‐metallic NH_4_^+^ ions in carbonyl/pyrazine sites achieved a two‐step 12e^−^ H‐bonding chemistry (Figure 4c), endowing QA‐COFs electrode with a high capacity of 220.4 mAh g^−1^ at 0.5 A g^−1^. This capacity was roughly twice those of metallic ions (Li^+^: 108.4 mAh g^−1^, Na^+^: 125.9 mAh g^−1^, K^+^: 71 mAh g^−1^). Theoretical and experimental results further revealed that the higher redox potential is related to NH_4_^+^ solvation behavior, originating from loose solvation structure, low desolvation hurdle, and flexible H‐bond network. The topo‐coordinated H‐bonding chemistry of NH_4_^+^ storage could be leveraged to design various organic electrode materials for ZOBs. In addition to COFs, polyimide materials have been reported as effective NH_4_^+^‐storage hosts, involving 1,4,5,8‐naphthalenetetracarboxylic dianhydride‐derived polyimide (PNTCDA), polyimide/nitrogen‐doped carbon/carbon nanotubes (PI/NDC) composite, and poly(1,4,5,8‐naphthalenetetracarboxylic anhydride naphthylamine) imine (PNNI).^[^ ^43^ ^]^ However, these materials still suffer from insufficient capacity due to the increase of inactive nodes, limited cycle lifespan resulted from material dissolution and/or manufacture complexity.

The structural originality of NH_4_^+^ inspires us to consider whether NH_4_^+^ can form H‐bonding networks with small organics, thereby stabilizing soluble matters and realizing durable capacity storage. Most recently, our group proposed knitting superstable lock‐and‐key H‐bonding networks between NH_4_^+^ ions and 2,7‐dinitropyrene‐4,5,9,10‐tetraone (DNPT) compound through H‐bonds.^[^ ^24^ ^]^ The octuple‐active carbonyl/nitro motifs of DNPT (H‐bond acceptor) were redox‐exclusively coordinate with tetrahedral flexible NH_4_^+^ charge carriers (H‐bond donator) through quadruple H‐bonds (N─H─O) but preclude rigid and larger Zn^2+^ ions (Figure 4d–f), owing to an ultralow activation energy (0.14 eV of ammoniation versus 0.31 eV of zincification). The multiple H‐bonding topo‐coordinated chemistry achieved fast diffusion kinetics of NH_4_^+^ ions, and conquered the stability barrier of DNPT in the electrolyte. Thus, high‐kinetics NH_4_^+^ coordination with DNPT cathode achieved stable two‐step 4e^−^ NH_4_^+^ charge storage (Figure 4g), contributing to a high‐rate capability (50 A g^−1^), a high capacity (320 mAh g^−1^), and an extraordinary lifespan (60 000 cycles) for Zn||DNPT cell (Figure 4h). This finding would represent a new paradigm for H‐bond stabilized small‐molecule organics to construct long‐life ZOBs.

In view of the structural and functional originality of H^+^ and NH_4_^+^ ions, their co‐coordination within organics is expected to deliver a synergistic vitality to conquer the kinetics and stability barriers of traditional Zn^2+^ storage, thereby achieving fast and durable energy storage. Very recently, for the first time, our group report NH_4_^+^/H^+^ co‐storage in self‐assembled organic superstructures (OSs) (Figure 4i),^[^ ^44^ ^]^ triggered by the interactions between benzoquinone and 2, 6‐diaminoanthraquinone through H‐bonding and π─π stacking. Benefiting from well‐defined superstructures, exposed carbonyl motifs and electron delocalization routes, OSs are redox‐exclusively coordinated with high‐kinetics NH_4_^+^/H^+^ but exclude rigid and sluggish Zn^2+^ ions. Systematic studies suggested the 4e^−^ NH_4_^+^/H^+^ charge storage mechanism (Figure 4j), which endowed OSs cathode with high‐rate capacity (157 mAh g^−1^ at 100 A g^−1^) and ultralong lifespan (50 000 cycles). Besides, OSs can be further electrochemically improved by modulating the organic building units. Despite significant efforts being made, the field is still in its infancy. Other types of potential organic electrode materials need further explored to design better NH_4_^+^‐based ZOBs.

### 3.2 Non‐Metallic Anion Storage

#### 3.2.1 Cl^−^ Ions as charge carriers

Chloride ions (Cl^−^) were first used as charge carriers in non‐aqueous batteries, but the choice of appropriate electrodes for Cl^−^ storage is challenging in aqueous electrolytes.^[^ ^45^ ^]^ This is because electrode chlorination induces the formation of chloride salts, which often dissolve in aqueous electrolytes and thus deteriorate electrochemical stability.^[^ ^46^ ^]^ Design of organic materials with redox‐active p‐type binding sites is expected to form powerful bonding forces with Cl^−^ charge carriers to inhibit solubility, but it is still ongoing.

Jiang group designed and synthesized a series of polypyrene cathode materials for Cl^−^ storage in ZOBs.^[^ ^47^ ^]^ Comparative studies unraveled that the linking pattern on the pyrene unit was pivotal to the electronic structure and redox activity of polypyrenes. The cross‐linked polypyrene with optimal 1,3,6,8‐linking pattern (CLPy) delivered highly porous structure, extended conjugation and favorable energy levels, triggering a reversible p‐type Cl^−^ doping reaction. As a result, Zn||polypyrene battery harvested an optimized capacity of 180 mAh g^−1^, which was much higher than other linear counterparts (24 and 44 mAh g^−1^). Significantly, the cell displayed an excellent cycling ability with 96.4% capacity retention after 38 000 cycles. Such outstanding electrochemical performances implied that polypyrene could be a promising cathode material for efficient Cl^−^ storage.

**Figure 5:**
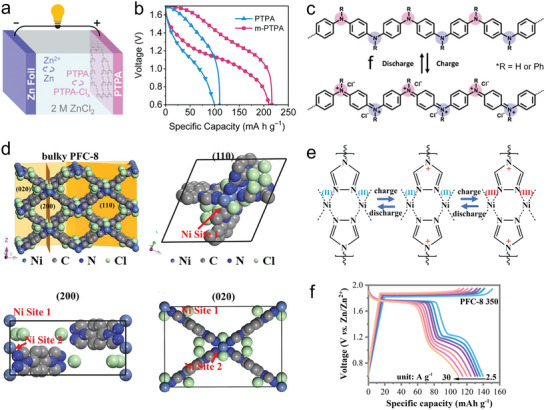
a) Working mechanism and b) voltage‐capacity curves of Zn||PTPA batteries. c) Charge storage mechanism of m‐PTPA cathode. Reproduced with permission.^[^ ^48^ ^]^ Copyright 2021, Wiley‐VCH. d) Atomic structures with various exposed crystal facets of PFC‐8 350. e) Illustration of two‐step redox reactions of PFC‐8 350 cathode. f) Voltage‐capacity profiles of Zn||PFC‐8 350 battery. Reproduced with permission.^[^ ^53^ ^]^ Copyright 2022, Wiley‐VCH.

The random folding characteristics of polymers often result in the active‐site underutilization and thus impair their electrochemical performances. Lu group designed a Cl^−^‐storage microporous conjugated polymer (m‐PTPA) by integrating tris(4‐aminophenyl)amine and tris(4‐bromophenyl)amine monomers periodically.^[^ ^48^ ^]^ When working as the cathode of a Zn battery, the 3D conjugated COF‐like m‐PTPA skeleton with rich amine species activated a pseudocapacitive‐dominated energy storage, accompanied by reversible Zn^2+^ plating/stripping process on the Zn‐metal anode (**Figure** **5**a). Significantly, m‐PTPA ensured high utilization of nitrogen redox‐sites (83.2% at 0.5 A g^−1^) and an ultrahigh energy density of 236 Wh kg^−1^ (Figure 5b). It was found that the charge storage mechanism of m‐PTPA cathode entailed high‐kinetics Cl^−^ uptake onto positively charged amine/imine motifs to form N─Cl^−^ bonds (Figure 5c). Although preliminary efforts have been made on Cl^−^ storage, further explorations of advanced polymer materials are essential toward the construction of better ZOBs.

Alternatively, bromide (Br^−^) and iodide (I^−^) anions, as redox‐active species with low toxicity, high abundance and fast reaction kinetics, can also be stored in ZOBs through conversion reactions on organic cathodes to realize efficient charge storage.^[^ ^49^ ^]^ As a proof‐of‐concept, Liu et al. designed Zn‐Br batteries constructed with the exfoliated covalent organic framework (exCOF) cathode, Zn anode and aqueous ZnBr_2_ electrolyte.^[^ ^50^ ^]^ As‐prepared porous functional exCOF immobilized soluble Br species via physical confinement and strong chemical adsorption, effectively facilitating the bidirectional conversion of polybromide. Consequently, Zn‐Br cell achieved high Coulombic efficiency of 99%, high capacity of 261 mAh g^−1^, and long cyclic life of 1000 cycles. This work would demonstrate the utilization potential of bromide‐based ZOBs. Besides, Yang et al. reported 2D porphyrin coordination supramolecular networks (Zn‐TCPP) as the cathode for aqueous iodine dual‐ion batteries.^[^ ^51^ ^]^ Systematic characterizations unraveled the charge‐transfer interaction between I_3_^−^ and porphyrin‐N, and confirmed the penetration effect of the layered network structure of Zn‐TCPP on I_3_^−^ species. Such a favorable electrochemistry contributed to desirable performances including high capacity (278 mAh g^−1^), high Coulombic efficiency (98%), high energy density (340 Wh kg^−1^) and long‐term cycling life (5000 cycles). These studies would harbinger the potentials of halogen‐based Zn batteries for advanced energy storage.

#### 3.2.2 OH^−^ Ions as Charge Carriers

In alkaline aqueous solutions, H^+^ concentration is very low, following the dynamic equilibrium constant of H_2_O dissociation.^[^ ^52^ ^]^ It is worth exploring whether OH^−^ ions can interact with surface redox‐active sites of organic electrodes via oxidation reaction during charging. Recently, Zhi group proposed a crystal‐facet engineering route to design metal‐organic frameworks (MOF)‐based cathodes with superior OH^−^ uptake capability for affording fast electrochemical kinetics.^[^ ^53^ ^]^ A thermally‐modified strategy was demonstrated to regulate the dominated facet (110) of Ni‐based MOF (PFC‐8 350) into optimal (020) and (200)‐rich exposing facets for supplying extra Ni sites in each asymmetric unit (Figure 5d). With strong OH^−^ adsorption behaviors and optimized electronic structures, both Ni and N species of PFC‐8 as the active sites achieved Ni^3+^/Ni^2+^ and N^+^/N^0^ redox reactions in the repeating coordination units (Figure 5e). Such electrochemical behaviors triggered desirable rate capability (110 mAh g^−1^ at 30 A g^−1^, Figure 5f) and excellent power density (42.7 kW kg^−1^). This research would open new doors to boost the electrochemical performances of MOF‐based ZOBs by the exposing crystal‐facet engineering.

Except for MOFs, COFs are also promising organic cathode materials due to their designable active groups, high crystallinity, and periodic skeletons. However, COFs materials are often trapped by their instability in harsh alkaline (OH^−^) chemical environment, triggering irreversible capacity loss after cycling. Thus, there leaves a large space for strategically improving the alkali tolerance of COFs by rational structural design. Zhi group reported a COF electrode (HPP‐COF) containing pyrazine and phenylimino groups for promoting ultra‐fast OH^−^ transportation by Grotthuss mechanism.^[^ ^54^ ^]^ The robust covalent linkage and H‐bond network between H_2_O molecules and phenylimino groups synergistically boosted the alkaline tolerance of HPP‐COF, endowing HPP‐COF electrode with excellent cyclic stability (30 000 cycles at 30 A g^−1^). This work would provide a new path for the structural design of COFs toward OH^−^‐storage ZOBs.

#### 3.2.3 Polyatomic Anions (CF_3_SO_3_^−^, SO_4_^2−^) as Charge Carriers

**Figure 6:**
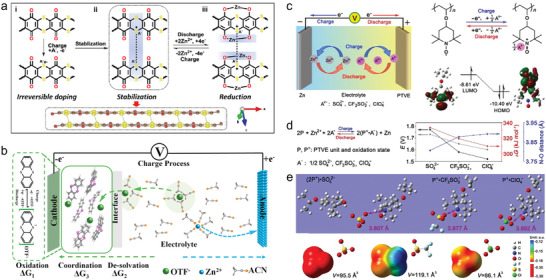
a) Electrochemical redox mechanism of PDB cathode. Reproduced with permission.^[^ ^55^ ^]^ Copyright 2020, Elsevier. b) Schematic reaction mechanism of TT cathode during the electrochemical process. Reproduced with permission.^[^ ^56^ ^]^ Copyright 2022, Wiley‐VCH. c) Schematic configuration of Zn||PTVE battery and redox states of PTVE with anions. d) Electrochemical reactions and voltages of Zn||PTVE battery. e) Optimized structures of anion‐coupled PTVE units and electrostatic potentials of various anions. Reproduced with permission.^[^ ^57^ ^]^ Copyright 2020, Wiley‐VCH.

Regulating the electrochemical performances of ZOBs by various polyatomic anion species is highly feasible, due to the stronger electrostatic interactions of polyatomic anions with organic radicals and thus the higher operation voltages. Zhang group proposed a bipolar redox chemistry of a ladder‐like poly(2,3‐dithiino‐1,4‐benzoquinone) cathode (PDB), which involved the anion delocalization of sulfur‐containing conjugated heterocyclic dithioether rings and the cation localization of benzoquinone units (**Figure** **6**a).^[^ ^55^ ^]^ An irreversible p‐type CF_3_SO_3_^−^‐doping first occurred in PDB cathode, forming intermolecular S─S interactions on the conjugated thioether (C─S─C) groups to further stabilize PDB molecules. Subsequently, reversible Zn^2+^ reaction with C═O group dominated the whole electrochemical processes during battery operation. Profiting from the unique redox process, PDB cathode delivered an ultrahigh energy density (190.1 Wh kg^−1^), a fast‐charge ability (30.6 s) and a high cycle life (10 000 cycles). Such an irreversible electrolyte anion‐doping redox chemistry would open a new door to design highly stable and long‐life ZOBs.

High‐voltage organic compounds are rare for the construction of high‐performance ZOBs. Benefiting from the structural stability caused by van der Waals and π─π stacking interactions, three p‐type organosulfur molecules including phenothiazine (PT), phenoxathiine (PX), and thianthrene (TT) molecules, were studied as the cathodes of ZOBs (Figure 6b).^[^ ^56^ ^]^ It was found that the electron cloud density of p‐type organics determined the voltage. A more uniform electron cloud distribution would result in a more difficult electron‐gaining/losing capability, which worked together with an improved desolvation energy to boost battery voltage. Electron cloud regulation and solvation structure manipulation enabled a high‐voltage (1.7 V) Zn||TT battery in an acetonitrile‐based electrolyte. Spectral results indicated that the S atoms in TT molecules acted as redox‐sites to reversibly coordinate with CF_3_SO_3_^−^ anions. These findings would pave a way to improve the work voltage of organic cathode materials for the practical applications of ZOBs. Despite its excellent properties, relying on organic electrolytes would lose some of the merits of aqueous electrolytes, such as low cost and safety.

Li and co‐workers studied the anion effects on the electrochemical properties of poly(2,2,6,6‐tetramethylpiperidinyloxy‐4‐yl vinyl ether) (PTVE) cathode in ZOBs (Figure 6c).^[^ ^57^ ^]^ CF_3_SO_3_^−^, SO_4_^2−^, and ClO_4_^−^ ions with different electrostatic potentials were selected as anion models. The stronger electrostatic interaction between anions and organic radicals led to a higher working voltage of ZOBs (Figure 6d). After the optimization of anion species, the well‐delocalized electronic structure of CF_3_SO_3_^−^ promoted its dissociation from PTVE cathode (Figure 6e), resulting in high electrochemical reversibility and stability. Zn||PTVE cell using Zn(CF_3_SO_3_)_2_ electrolyte thus exhibited excellent power density (14 kW kg^−1^) and good cyclic life (1000 cycles), showing high fast‐charging stability. These results would bring new insights into the anionic chemistry of p‐type organics and inspire more explorations into the anion effect toward advanced battery design.

### 3.3 Non‐Metallic Anion/Cation Co‐Storage

Thanks to the structural designability of organic materials, different n‐type and p‐type active motifs can be integrated into redox‐bipolar frameworks, where cation and anion storage can independently occur on the electroactive organic sites. Compared with the well‐known single‐ion storage mechanism with Coulomb repulsion between same ions, the co‐storage of opposite ions in ZOBs gives enhanced electrochemical metrics.^[^ ^58^ ^]^ This is because the cation‐anion Coulomb interactions in organic skeletons can significantly promote reaction kinetics by rejuvenating fast ion‐migration toward electrode interiors. Developing bipolar organic host materials for ion co‐storage offers new opportunities to propel ZOBs.

**Figure 7:**
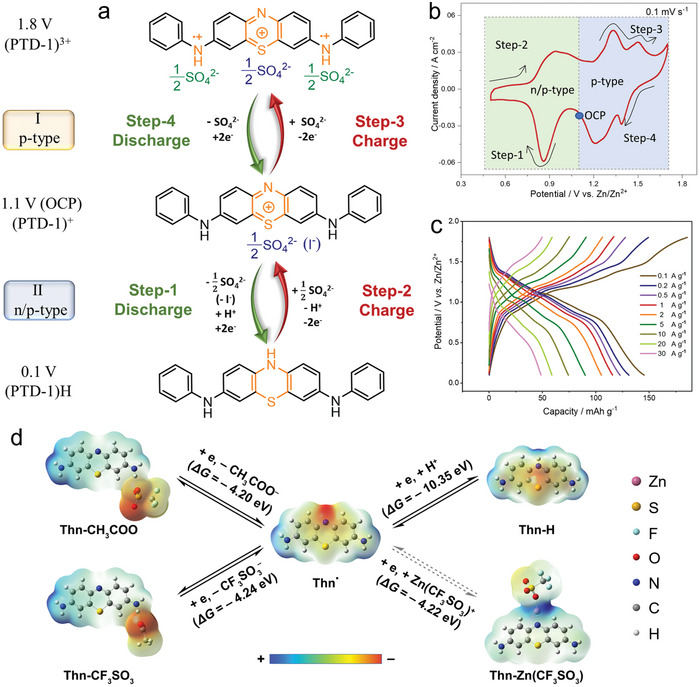
a) Redox reaction mechanisms, b) CV profile and c) voltage‐capacity curves of PTD‐1 cathode in aqueous ZnSO_4_ electrolyte. Reproduced with permission.^[^ ^29^ ^]^ Copyright 2021, Wiley‐VCH. d) Redox reaction mechanisms, geometrical structures, electrostatic potentials, and Gibbs free energy changes (*ΔG*) of Thn‐CH_3_COO. Reproduced with permission.^[^ ^59^ ^]^ Copyright 2023, Wiley‐VCH.

Wang group reported a bipolar bis(phenylamino)phenothiazin‐5‐ium iodide (PTD‐1) organic cathode for ZOBs to tap into the synergetic advantages of n‐type and p‐type redox reactions in ZnSO_4_ electrolyte.^[^ ^29^ ^]^ Experimental and theoretical results first clarified the hybrid 4e^−^ redox charge storage of PTD‐1 cathode (**Figure** **7**a), involving the n/p‐type redox reaction of two phenylimino groups at low potential (i.e., H^+^, SO_4_^2−^ storage) and p‐type redox reaction of phenothiazine heterocyclics at high potential (i.e., SO_4_^2−^ storage) (Figure 7b). This mechanism could make full utilization of the abundant electrolyte ions, which was different from traditional inorganic materials and single n‐type or p‐type organic materials that only stored cations or anions. As a result, Zn//PTD‐1 battery harvested a capacity of 188.24 mAh g^−1^, an average voltage of 1.1 V, and a high energy density of 116.83 Wh kg^−1^ (Figure 7c). Besides, an organic biological dye, phenothiazine‐based thionin (Thn‐CH_3_COO) also exhibited a bipolar redox process in Zn(CF_3_SO_3_)_2_ electrolyte,^[^ ^59^ ^]^ involving p‐type CH_3_COO^−^/CF_3_SO_3_^−^ insertion and n‐type H^+^ insertion (Figure 7d). All these results demonstrated the flexible design of redox‐bipolar organic materials and would broaden the mechanism understandings for the further development of ZOBs.

### 3.4 Performances of Non‐Metallic Ion Batteries

Battery study is performance‐oriented. Metallic Zn^2+^ ion has been considered as the default charge carrier, while the advantages of non‐metallic charge carriers are gradually being explored to continuously optimize ZOBs, mainly focusing on boosting specific capacity (*C*_m_), average discharge voltage (*V*_average_), energy density (*E*), rate capability and cycling lifespan (**Table** **2**). *E* is the product of *C*_m_ and *V*_average_. The rate capability reflects the fast‐charging ability of electrodes at different currents, which largely depends on the reaction kinetics of non‐metallic charge carriers within organic hosts. Besides, cycle life is related to the structural robustness of organic materials during the repeated (dis)charging process. Here, the characteristics of various non‐metallic charge carriers applied in ZOBs are systematically summarized and compared, suggesting their great prospects for energy‐related applications.

**Table 2:** Performance comparison of various non‐metallic ion storage in ZOBs.

Organic Structure	Charge Carrier	*C*_m_ [mAh g^−1^]	*V*_average_ [V]	*E* [Wh kg^−1^]	Capacity Retention [%]	Ref.
HATN	H^+^	405@0.1 A g^−1^ 123@20 A g^−1^	≈0.55	N/A	93.3%, 5000 cycles, 5 A g^−1^	[17]
TABQ	H^+^	303@0.1 A g^−1^ 213@5 A g^−1^	≈0.8	N/A	83%, 1000 cycles, 5 A g^−1^	[30a]
BQPH	H^+^	429@0.1 A g^−1^ 145@20 A g^−1^	≈0.8	N/A	82%, 1000 cycles, 10 A g^−1^	[32]
HBOSs	H^+^	311@1 A g^−1^ 135@150 A g^−1^	0.86	267	92.3%, 50 000 cycles, 10 A g^−1^	[39]
PO	H^+^	310@0.1 A g^−1^ 98@20 A g^−1^	0.6	N/A	95%, 400 cycles, 0.2 A g^−1^	[13]
OEM	H^+^	284@0.05 A g^−1^ 163@0.8 A g^−1^	0.75	120	95%, 200 cycles, 5 A g^−1^	[35]
HOF‐HATN	H^+^	320@0.05 A g^−1^ 100@10 A g^−1^	≈0.55	N/A	88%, 10 000 cycles, 5 A g^−1^	[31b]
QA‐COFs	NH_4_^+^	220.4@0.5 A g^−1^ 37.5@10 A g^−1^	N/A	N/A	100%, 7000 cycles, 6 A g^−1^	[11c]
PNTCDA	NH_4_^+^	150@0.1 A g^−1^ 110.3@5 A g^−1^	N/A	68.7	80.2%, 10 000 cycles, 5 A g^−1^	[43a]
DNPT	NH_4_^+^	320@0.2 A g^−1^ 113@50 A g^−1^	0.71	226	81.2%, 60 000 cycles, 10 A g^−1^	[24]
OSs	H^+^ NH_4_^+^	299@1 A g^−1^ 157@100 A g^−1^	0.56	166	82.8%, 50 000 cycles, 10 A g^−1^	[44]
CLPy	Cl^−^	180@0.05 A g^−1^ 105@3 A g^−1^	≈0.9	N/A	96.4%, 38 000 cycles, 3 A g^−1^	[47]
m‐PTPA	Cl^−^	211@0.5 A g^−1^ 108@6 A g^−1^	≈1.1	236	87.6%, 1000 cycles, 6 A g^−1^	[48]
PFC‐8 350	OH^−^	139@2.5 A g^−1^ 110@30 A g^−1^	≈1.5	211.9	77.2%, 950 cycles, 10 A g^−1^	[53]
HPP‐COF	OH^−^	184@5 A g^−1^ 91.7@100 A g^−1^	≈0.85	155.8	79.1%, 30 000 cycles, 30 A g^−1^	[54]
PDB	OTF^−^	205@0.05 A g^−1^ 176@10 A g^−1^	≈0.9	190.1	75%, 10 000 cycles, 20 A g^−1^	[55]
TT	OTF^−^	112@0.5 A g^−1^ 66@20 A g^−1^	≈1.1	≈120	82%, 8000 cycles, 1 A g^−1^	[56]
PTVE	OTF^−^	83@0.2 A g^−1^ 52@10 A g^−1^	1.58	≈125	77%, 1000 cycles, 1 A g^−1^	[57]
PTD‐1	H^+^ SO_4_^2−^	188@0.04 A g^−1^ 48.9@30 A g^−1^	1.1	116.8	82.5%, 4000 cycles, 1 A g^−1^	[29]
Thn‐CH_3_COO	H^+^ OTF^−^	162@0.1 A g^−1^ 113@5 A g^−1^	≈0.75	≈120	65%, 500 cycles, 1 A g^−1^	[59]
MnO_2_	Zn2+	192.4@0.5	≈1.3	≈250	88.9%, 600 cycles, 0.7 A g−1	[76b]
δ‐MnO_2_	Zn2+	238@0.2C 138@20C	≈1.3	≈300	93%, 4000 cycles, 20 C	[76c]
V_2_O_5_	Zn2+	160@1 A g−1 100@5 A g−1	≈0.8	≈128	90.1%, 16 000 cycles, 2 A g−1	[76d]
Zn*x*V_2_O_5_·nH_2_O	Zn2+	373.7@0.3 A g−1 ≈150@2 A g−1	≈0.7	≈260	87.3%, 1000 cycles, 0.5 A g−1	[76e]
Prussian blue	Zn2+	108.3@1 A g−1 67.5@8 A g−1	1.75	≈210	99.3%, 5000 cycles, 5 A g−1	[76f]

#### 3.4.1 Specific Capacity

Organic materials with high‐density redox‐sites and resulting high theoretical capacity are the primary prerequisite for improving the output capacity of ZOBs, according to the equation: *C*_m_ = *nF*/3.6 *M* (*n* is the electron transfer number, *F* is the Faraday constant, and *M* is the molar mass of organic materials). Using the same redox type of cathodes, the storage of cationic non‐metallic charge carriers realizes capacities comparable and even higher than that of metallic Zn^2+^ ion. This is because they have small hydrated structure and low diffusion barrier at the electrode/electrolyte interface, which does a favor to efficient charge storage, especially at large current densities.^[^ ^11a^ ^]^ The actual capacities of ZOBs at low current rates mainly rely on organic cathodes, which are usually restricted by the factors of pore parameters, geometry and electronic conductivity as well as electrolytes, although there often has a trade‐off between them.

Boosting the porosity and surface area of organic materials by microstructure regulation, can empower fast ion migration and expose more active sites, thus enhancing electrochemical capacities.^[^ ^60^ ^]^ For example, m‐PTPA cathode possessed a much higher surface area (655 m^2^ g^−1^) than that of PTPA (32 m^2^ g^−1^) because of its microporous structure.^[^ ^48^ ^]^ It thus displayed a higher capacity of 210.7 mAh g^−1^ at 0.5 A g^−1^ than PTPA (99.8 mAh g^−1^). Further, the conjugated molecular engineering (e.g., poly(2, 9‐dihydroquinoxalino[2, 3‐b]phenazine) (PO) molecule)^[^ ^13^ ^]^ and the incorporation of organics into graphene oxide (e.g., graphene/aza‐fused π‐conjugated microporous polymer)^[^ ^61^ ^]^ could be effective routes to substantially enhance the capacity of organic materials. Significantly, organic superstructures (e.g., microflowers, nanoarrays) with more exposed high‐redox‐active motifs and high‐speed electron transfer routes have exhibited competitive capacities (310 mAh g^−1^ at 1 A g^−1^).^[^ ^39^ ^]^ Exploration of organic superstructures represents an emerging research direction for ZOBs. Besides, excessive or too low concentration of various non‐metallic charge carriers in electrolytes could bring distinct capacities of ZOBs. Therefore, optimizing electrolyte formula is also very important for designing high‐capacity ZOBs.

#### 3.4.2 Average Discharge Voltage

Voltage is another important factor affecting the energy density of ZOBs, determined by both electrodes and electrolytes. P‐type organics with positive valence conversion are considered to have higher operation voltage than n‐type organics. Based on thermodynamics, the voltage (*E*) can be calculated through the form: *E* = −Δ*G*/*nF* (Δ*G*, *n* and *F* are the change of Gibbs free energy, electron transfer number and Faraday constant, respectively).^[^ ^22d^ ^]^ In principle, boosting the value of Δ*G*/*n* could lead to higher output voltage. For example, in the case of Zn||PTVE battery, theoretical calculation suggested that the Δ*G*/*n* of 2(PTVE^+^)SO_4_^2−^ was larger than those of PTVE^+^CF_3_SO_3_^−^ and PTVE^+^ClO_4_^−^. As a consequence, the voltage of Zn||PTVE cell in ZnSO_4_ electrolyte was superior to the cases in Zn(CF_3_SO_3_)_2_ and Zn(ClO_4_)_2_ electrolytes.^[^ ^57^ ^]^

The voltages of ZOBs can also be regulated by molecular engineering of organic cathodes. The electronic inductive effect of side groups determines the energy state and electrochemical response of organics.^[^ ^62^ ^]^ As well‐established, a lower level of lowest unoccupied molecular orbital (LUMO) signifies a higher electron affinity, contributing to a higher redox voltage.^[^ ^63^ ^]^ The introduction of electron‐withdrawing groups (e.g., ─CN, ─F, ─Cl, ─Br) can reduce the LUMO energy level of organics to boost their voltages, whereas electron‐donating groups (e.g., ─CH_3_, ─OCH_3_, ─NH_2_, ─OH) exhibit the opposite effect.^[^ ^16^, ^64^ ^]^ For example, poly(5‐cyanoindole) reached a higher discharge plateau of 1.35 V in ZOBs by introducing ─CN group to polyindole.^[^ ^65^ ^]^ Furthermore, the position of redox‐active groups could also affect the discharge voltage of ZOBs, as reflected by the higher voltage of *para*‐dinitrobenzene cathode compared to *meta‐* and *ortho*‐position dinitrobenzene.^[^ ^34^ ^]^

Briefly, high‐energy‐density ZOBs could be created through manipulating the key features of organic cathode materials (electron‐acceptor sites, microstructures, electron structures and side‐group effects), as well as rationally optimizing the types of non‐metal ionic carriers in electrolytes, thereby substantially revolutionizing high capacity and high‐voltage charge storage.

#### 3.4.3 Rate Capability

The rate performance of the battery that reflects its fast‐charging ability is particularly important for the energy conversion of intermittent power sources. Metallic Zn^2+^ storage in organic materials such as poly(phenazine‐alt‐pyromellitic anhydride) (PPPA),^[^ ^6^ ^]^ π‐d conjugated metal‐organic framework,^[^ ^8b^ ^]^ benzothiadiazole‐functionalized olefin‐linked COF (COF‐TMT‐BT),^[^ ^66^ ^]^ hydroquinone‐based COF (HqTp),^[^ ^67^ ^]^ and quinoxalinophenazinedione covalent triazine framework (CTF‐TTPQ),^[^ ^68^ ^]^ usually delivered rate capabilities of <5 A g^−1^, making it less suitable for fast rechargeability. In stark contrast, non‐metallic charge carriers often enable rapid surface redox reactions to increase the rate capability of electrodes, of which small‐sized non‐metallic ions contribute to ultrahigh‐rate capability. For example, H^+^ storage through rapid Grotthuss proton conduction within H‐bonded superstructure networks delivered an outstanding large‐current viability of 150 A g^−1^, which was the fastest among ZOBs.^[^ ^39^ ^]^ NH_4_^+^ storage also enables high‐rate capabilities of 10–50 A g^−1^, depending on the organic electrode materials. Due to large ionic sizes and strong bonding forces, non‐metallic anions (e.g., CF_3_SO_3_^−^, SO_4_^2−^, Cl^−^) generally follow diffusion‐limited kinetics, leading to moderate rate performances (5–20 A g^−1^).

Except for charge carriers, the redox kinetics of ZOBs is also affected by the intrinsic conductivity of organic materials.^[^ ^69^ ^]^ The inferior electronic conductivity of organics usually results in unsatisfactory rate performance, which can be improved by extending π‐conjugated organic units and encapsulating organics within carbon skeletons.^[^ ^9^, ^70^ ^]^ The extended π‐electron conjugation in redox‐active compounds triggers enhanced π‐electron delocalization to promote electron migration rate of organic materials.^[^ ^71^ ^]^ For instance, BQPH with a large π‐conjugated planar structure showed a superior rate viability of 20 A g^−1^ than that of tetraamino‐*p*‐benzoquinone (TABQ, 5 A g^−1^).^[^ ^30^, ^32^ ^]^ Besides, large‐surface‐area and high‐conductivity nanostructured carbons can act as ideal hosts of organics, giving easily electron transfer routes.^[^ ^72^ ^]^ After incorporating *para*‐dinitrobenzene into carbon nanoflower, the composite cathodes delivered high capacities of 136–214 mAh g^−1^ even at 20 A g^−1^, while very limited capacities of 10–37 mAh g^−1^ could be obtained for pure organic molecule.^[^ ^34^ ^]^

Briefly, high‐rate ZOBs could be achieved through the rational design of redox‐active organic cathode materials with π‐conjugated aromatic structures and high electron conductivities, and the rational use of non‐diffusion‐controlled high‐kinetics H^+^/NH_4_^+^ ions, for maximizing the spatial capacitive charge storage density even at large current rates.

#### 3.4.4 Cycling Life

Cycling life is mainly related to the structural stability of organic cathodes, while non‐metallic cationic carriers usually cause less structural variation due to their small hydrated size.^[^ ^73^ ^]^ Non‐metallic cation‐based ZOBs generally deliver superior capacity retention or cyclic stability than those of anion and Zn^2+^‐based ones. Such differences can be attributed to the high charge density and large energy penalty of Zn^2+^ ions, which results in strong electrostatic interactions with organic electrodes,^[^ ^9^, ^66^ ^]^ triggering irreversible structural change and dissolution of electrodes. Cyclic irreversibility of non‐metal ions and dissolution problem of organic cathodes often leads to capacity loss during (dis)charging cycles. Significantly, the favorable interactions between non‐metallic charge carriers and organic cathode materials, such as Grotthuss H^+^ conduction and NH_4_^+^‐coordinated H‐bonding chemistry, can enable ultrastable and rapid cycling. Thus, inhibiting the dissolution of organics and boosting their non‐metallic ion‐storage ability are the priority task for extending the life of ZOBs. Besides, the protection of Zn anodes and the optimization of electrolytes also are important factors for the cycling stability of ZOBs, which deserve further attention.

A robust triangular phenanthrenequinone‐based macrocycle structure was designed to deliver a low solubility in aqueous electrolyte and thus achieved an extended cycling stability without obvious capacity degradation.^[^ ^9^, ^74^ ^]^ In addition to molecular engineering, encapsulating organic molecules in conductive carbon skeletons can also suppress the hydrolysis of active species due to the π‐π interaction between organics and carbons. For example, *para‐*dinitrobenzene was encapsulated in carbon nanoflowers to form insoluble composite, which showed superior cycle life of 25 000 cycles at 5 A g^−1^.^[^ ^34^ ^]^ Besides, compared with organic small molecules, polymers also display enhanced anti‐dissolution and structural robustness due to their covalent structures.^[^ ^75^ ^]^ However, the twisted molecule chains and random folding traits of polymers often cause difficulties in full utilization of active redox‐sites.

3D organic superstructures with more exposed active motifs and high‐speed electron transfer routes show promising prospects for energy realms. Especially, the construction of H‐bonding organic superstructure networks via intermolecular H‐bonds was particularly suitable for ultrafast Grotthuss H^+^ storage and could suppress the solubility of active materials, thus afford ZOBs with the highest number of cycles ever reported (up to 50 000 cycles at 10 A g^−1^).^[^ ^39^ ^]^ Besides, the structural originality of NH_4_^+^ ions show a strong vitality to coordinate with DNPT to form ultrastable lock‐and‐key H‐bonding networks, thereby stabilizing soluble molecules to push the service life of ZOBs to a new level (60 000 cycles at 10 A g^−1^).^[^ ^24^ ^]^ Besides, long‐term cyclic performance is generally evaluated under large currents, where the impact of side reactions is minimal. The evaluation of cycling stability at low currents is related to the reaction potentials located near HER/OER potentials, as they may be switched on at low charging rates.

Briefly, long‐life ZOBs could be created by extending π‐conjugated organic active‐units, introducing insoluble encapsulation scaffold, and polymerizing soluble monomers, but new problems arise including manufacture complexity, the increase of inactive nodes and low utilization of active sites. This is not the case for non‐metallic Grotthuss H^+^ conduction and H‐bonding NH_4_^+^ chemistry in well‐designed organic materials, which represent hopeful research directions for the achievement of advanced ZOBs in the future.

Inorganic materials such as manganese‐based compounds, vanadium‐based oxides and Prussian blue analogs are also extensively studied as the cathode for zinc batteries, due to their high energy density, appropriate redox voltage, and multielectron reaction.^[^ ^3^ ^]^ However, repeated insertion/extraction of Zn^2+^ ions with high electrostatic repulsion often causes sluggish reaction kinetics and large structural disturbances in inorganic compounds. Unlike inorganic materials, redox‐active organic materials store energy via surface coordination reactions, avoiding large structural damage.^[^ ^16^ ^]^ The fast and stable uptake/removal of non‐metallic ions in organic cathode materials substantially reform the comprehensive metrics of ZOBs in terms of rate capacity, voltage, energy density and cycling life, exceeding typical Zn//inorganic full batteries (Table 2).^[^ ^76^ ^]^ Besides, compared with the low reserves of inorganic metal elements, organic materials are composed of earth‐abundant non‐metal C/H/O/N elements,^[^ ^22d^ ^]^ highlighting the large‐scale application potential of ZOBs toward a greener energy world in the future.

Non‐metallic charge carriers also exert electrochemical effects on Zn anodes in ZOBs. For example, high‐kinetics H^+^ storage is crucial for advanced ZOBs, but its intrinsic acidic characteristics tends to corrode metal anodes,^[^ ^12^ ^]^ resulting in structural damage of Zn anode surfaces and unsatisfactory reversibility during the repetitive Zn plating/stripping process. In contrast, flexible NH_4_^+^ storage is much less corrosive, which helps to avoid the side reactions and establish a stable electrochemical environment for the operation of ZOBs.^[^ ^14^ ^]^ Besides, Cl^−^ ions in the high‐concentration zinc salt electrolyte can suppress the hydrolysis of Zn^2+^ to afford highly reversible and stable Zn metal anodes.^[^ ^47^ ^]^ Thus, it is vital to reasonably modulate the compatibility between Zn anodes and non‐metal ions in ZOBs. With the anode/ion/cathode interface chemistry under control, aqueous ZOBs are ideally suitable as large‐scale energy storage systems to achieve a greener rechargeable world.

Throughout the development course of ZOBs, compared with metallic Zn^2+^ storage, the electrochemical performances of non‐metallic charge carrier storage are significantly characterized by superior capacity, fast chargeability and long lifespan. In contrast to boosting the performances, the cost and scale of ZOBs seem to receive little attention. Low cost and excellent electrochemical performance are the two important indicators that determine the commercialization of ZOBs. The cost of ZOBs is mainly determined by organic materials and aqueous electrolytes, in addition to battery fabrication, maintenance, environmental impact and operation costs. In this regard, non‐metal‐ion electrolytes (e.g., H^+^ and NH_4_^+^) composing of Earth‐abundant elements can be applied to decrease the cost of electrolytes, as they are cheaper than metallic Zn^2+^ ion, especially in large amounts. Besides, some high‐performance organic materials are not cost‐effective due to their complex synthesis. Therefore, constant efforts will be needed to build more efficient, low‐cost and grid‐scale ZOBs through the rational design of multifunctional organic materials that integrate multi‐electron redox‐sites, favorable bipolar attributes and stable topologies, as well as the careful selection of high‐kinetics non‐metal charge carriers.

## 4 Summary and Outlook

Non‐metal ions have inspired growing interest for batteries due to their structural and dynamic merits. To meet the urgent requirements of advanced energy storage systems, a variety of non‐metallic ions are being explored to build advanced ZOBs. This review systematically summarizes the recent achievements of non‐metal ion storage in ZOBs, with a detailed categorization according to their physicochemical features and redox interactions with organic cathode materials. The application effectiveness of non‐metal‐ions ZOBs was also highlighted, which would offer useful framework for understanding the structure–performance relationships between organics and ionic carriers. Electrode structures can guide the selection of suitable non‐metal ions, and the redox mechanism between the two follows the donor–acceptor principle. Ionic size and desolvation energy of charge carriers in electrolytes, as well as the structure and function of organic electrodes all impact the metrics of ZOBs. Despite the significant advances made in recent years, some key aspects and opportunities for future research of ZOBs need to be focused on the following areas.

1. *Closely combining the favorable properties of non‐metal ions with organics to match the target requirements*. The key to further enlarging the special application circumstances of non‐metal ions is how to couple their unique structural and functional superiorities with organic electrodes. For example, the size and kinetics merits of H^+^ pose high demands for the controlled and on‐demand design of organic materials. How to realize precise regulation over the microstructures of organic materials at the nanoscale is the key issue to create consecutive H‐bonding networks for propelling ultrafast Grotthuss H^+^ conduction and storage. Another example is the use of tetrahedral NH_4_^+^ ions to coordinate organics to knit lock‐and‐key H‐bond networks, which overcomes the stability barriers of organics in electrolytes and provides rapid ion‐reaction kinetics. How to dig up the potentials of ZOBs with non‐metallic charge carriers is a development direction worth considering in current laboratory exploration. With increasing efforts devoted to this filed, we believe that non‐metal ions will continue to bring remarkable breakthroughs to ZOBs.
2. *Expanding the working voltage of non‐metal‐ion ZOBs*. Subjected to the thermodynamic stability of H_2_O molecules in electrolytes, ZOBs with non‐metallic ions exhibit relatively narrow electrochemical potential windows, which inhibits further increase in energy density and is thus challenging to be compensated by high capacity. The implement of concentrated additive solutions, “water‐in‐salt” or organic‐based electrolytes can be considered to extend and stabilize the voltage range of the battery through reducing the activity “free” water in the electrolyte. Of note, too high concentration of additives and excessive organic solvents would greatly decrease the amount of “free” water, but inevitably impair some beneficial characteristics of non‐metallic ions such as transport kinetics. Fortunately, safety is not lost even though the content of “free” water of the electrolyte is low. Overall, how to balance the content of “free” water and stable wide voltage window to maximize energy density is of great research significance for building ZOBs.
3. *In‐depth understanding of the ionic charge storage mechanism in ZOBs*. Although the redox reaction mechanism of organic electrodes is unraveled at the molecular level, the structural details and ion transfer pathways at the electrode/electrolyte interface are still elusive. The states of organic intermediates during ion (de)coordination need further exploration with the aid of advanced in situ characterization techniques such as cryo‐electron microscopy, X‐ray photoelectron spectroscopy, Fourier transform infrared spectra and X‐ray diffraction. The emerging artificial intelligence‐based machine learning methodologies are promising to speed up the screening of organic materials and charge carriers, and the analysis of structure evolutions and redox mechanisms of ZOBs in unknown states. This ensures the efficiency and accuracy of theoretical calculations, providing great potentials for data‐driven research in advancing state‐of‐health evaluation and governance of ZOBs under on‐demand working conditions. Marriage of updating characterizations and computational techniques is expected to witness the vigorous development of ZOBs in real uses in a near future.
4. *Scale applications of ZOBs with practical functions and performances*. The electrochemical metrics of ZOBs, even for full batteries and pouch‐type cells, are often tested under the conditions far from practical levels, which could not satisfy the industrial requirements. The large‐scale production and commercialization of high‐performance organic materials are mainly influenced by factors such as raw material cost, synthesis route, active matter loading, energy density and recyclability, etc. These factors are important parameters for the practical applications of organic materials in future energy storage devices. How to utilize green and low‐cost manufacturing technologies for large‐scale production and recyclability of organic matters at the cellular level will be an attractive issue. Besides, the actual design factors of the battery (safety/tolerance testing, areal mass loading, the amount of electrolyte, practical energy/power density, large‐scale integration, etc.) should be taken into consideration together when evaluating the electrochemical performances at pouch‐type cell levels.

Through the exploration of non‐metal charge carriers in electrolytes, the rational design of electrodes and the engineering process of cells, ZOBs have undergone vigorous development. However, there is still a long way to go before seeing the practical applications of ZOBs in the future energy world, especially in electric vehicles and high‐tech devices. Finding a relative balance between achievement, cost and sustainability would accelerate the commercialization process of ZOBs. Therefore, unremitting efforts will be needed to fulfill the superiorities of next‐generation ZOBs such as industry potential, sustainability, power supply, fast kinetics, tailor‐made function, high safety.

## Conflict of Interest

The authors declare no conflict of interest.
